# Only 8% of major preventable adverse events after hip arthroplasty are filed as claims: a Swedish multi-center cohort study on 1,998 patients

**DOI:** 10.1080/17453674.2019.1677382

**Published:** 2019-10-16

**Authors:** Martin Magnéli, Maria Unbeck, Bodil Samuelsson, Cecilia Rogmark, Ola Rolfson, Max Gordon, Olof Sköldenberg

**Affiliations:** a Department of Clinical Sciences at Danderyd Hospital, Division of Orthopaedics, Karolinska Institutet, Stockholm;; b Department of Neurobiology, Care Sciences and Society, Karolinska Institutet, Stockholm;; c Department of Clinical Sciences Malmö, Lund University Clinical and Molecular Osteoporosis Research Unit, Malmö;; d Department of Orthopaedics, Institute of Clinical Sciences, Sahlgrenska Academy, University of Gothenburg, Mölndal, Sweden

## Abstract

Background and purpose — Hip arthroplasty is one of the most performed surgeries in Sweden, and the rate of adverse events (AEs) is fairly high. All patients in publicly financed healthcare in Sweden are insured by the Mutual Insurance Company of Swedish County Councils (Löf). We assessed the proportion of patients that sustained a major preventable AE and filed an AE claim to Löf.

Patients and methods — We performed retrospective record review using the Global Trigger Tool to identify AEs in a Swedish multi-center cohort consisting of 1,998 patients with a total or hemi hip arthroplasty. We compared the major preventable AEs with all patient-reported claims to Löf from the same cohort and calculated the proportion of filed claims.

Results — We found 1,066 major preventable AEs in 744 patients. Löf received 62 claims for these AEs, resulting in a claim proportion of 8%. 58 of the 62 claims were accepted by Löf and received compensation. The claim proportion was 13% for the elective patients and 0.3% for the acute patients. The most common AE for filing a claim was periprosthetic joint infection; of the 150 infections found 37 were claimed.

Interpretation — The proportion of filed claims for major preventable AEs is very low, even for obvious and serious AEs such as periprosthetic joint infection.

In Sweden, cemented total hip arthroplasty was the 7th most performed surgery in 2016 (SPOR 2016), and over 20,000 are performed every year (Kärrholm et al. 2017). High-volume surgeries may generate a considerable number of adverse events (AEs). The AE rate following elective total hip arthroplasty ranges from 4.1% to 10% (Huddleston et al. 2012, Bohl et al. 2016, Richards et al. 2018).

The Swedish Patient Injury Act states that all healthcare providers are obliged to have insurance covering AEs (Swedish Parliament 2010). Additionally, providers are obliged to urgently inform patients who sustain an AE about their right to claim compensation (Swedish Parliament 2010). All publicly financed Swedish healthcare and most privately financed, regardless of whether the provider is public or private, is insured by Löf (“Landstingens Ömsesidiga Försäkringsbolag”, “the Mutual Insurance Company of Swedish County Councils”).

Patients who sustain an AE have the right to file a claim to Löf. The claim is assessed by experts at Löf and if the insurance terms are fulfilled, the patient will receive compensation. It is a non-fault insurance system, and there is no legal consequence for either the individual caregiver, or the healthcare provider.

Orthopedics is the specialty with the largest amount (28%, n = 1,492) of accepted AEs that received compensation in 2016 (Löf 2016). Considering the high number of hip arthroplasties and the incidence of AEs, there is reason to suspect that too few patients file insurance claims after AEs.

We assessed the proportion of patients who sustained a major preventable AE and filed a claim for compensation to Löf.

## Methods

### Study design

This is a national multi-center cohort study on data from medical records, insurance records and register data. It is part of a larger study on AEs after hip arthroplasty named VARA (Validation of Register Data after Hip Arthroplasty) (Magnéli et al. 2019). The exposure was major preventable AEs following hip arthroplasty within 90 days and the outcome was accepted claims to Löf.

### Setting

All patients aged 18 and older who received either a hemi- or total hip arthroplasty in 1 of 4 major county councils in Sweden (Stockholm, Skåne, Västra Götaland, and Västerbotten) during 2009–2011 and reported to the Swedish Hip Arthroplasty Register (SHAR) were eligible for inclusion in this study. The surgeries were performed in 24 different hospitals (6 university hospitals, 5 central county hospitals, 7 county hospitals, and 6 private hospitals reimbursed by the county councils). Almost all hip arthroplasties in Sweden are publicly financed and the patients are thereby insured by Löf. We included patients with both elective and acute surgeries.

### Study size and participants

The sample size was calculated for the VARA project, a project designed to validate a Swedish instrument for measuring AEs after hip arthroplasty. We used a weighted sample for increasing the chance of selecting patients with an AE, thereby avoiding excess record review.

20 different selection groups for acute and elective arthroplasties were created as follows (Table 1, see Supplementary data).

**Table 3.** Demographics for the different groups. Values are frequency (%) unless otherwise specified

Factor	All patients N = 1,998	Claim group n = 61	AE group n = 744	No AE group n = 1,254
Age, median	77	69	78	76
range	18–100	18–88	18–99	18–100
Female	1,250 (63)	35 (57)	458 (62)	792 (63)
Male	748 (37)	26 (43)	286 (38)	462 (37)
Elective surgery	1,331 (67)	56 (92)	470 (63)	861 (69)
Acute surgery)	667 (33)	5 (8)	274 (37)	393 (31)
Total arthroplasty	1,422 (71)	57 (93)	497 (67)	925 (74)
Hemiarthroplasty	576 (29)	4 (7)	247 (33)	329 (26)

AE = adverse event.

1. We constructed 3 arbitrary groups dividing patients on lengths of primary stay in percentiles divided as 0–55%, 56–80%, and 81–100%. The 3 groups were further divided based on whether there was an ICD-10 code (WHO 2017) indicating an AE in the National Patient Register (NPR) (Table 2, see Supplementary data). Overall, 6 groups were generated.
2. A selection was made for patients who had readmissions in the NPR. The readmission groups were divided in readmission within 2–30 days and within 31–90 days after surgery. The 2 groups were further divided based on whether there was an ICD-10 code indicating an AE in the NPR, generating 4 groups.

**Table 4.** Proportion of filed claims (%) for major preventable AEs

Factor	Proportion	CI
All patients	8	1–15
Acute patients	0.3	0.1–0.6
Elective patients	13	1–40
Elective/acute ratio	46	2–267

AEs = adverse events.

CI = 95% confidence interval limit.

This sampling process was repeated for both acute and elective patients, which resulted in 20 selection groups.

### Data sources

We recruited the study cohort from the SHAR that also supplied data on the primary surgeries. The completeness of the register is approximately 98% (Kärrholm et al. 2017).

We received the dates and type of clinic on all admissions and unplanned outpatient visits at the hospitals from the NPR. This data were used to track all admissions to be reviewed. The NPR has had total national coverage since 1987 (Ludvigsson et al. 2011, Socialstyrelsen 2016).

We linked data on the primary surgery from the SHAR to the NPR using the Swedish personal identity number as a unique identifier. With the crossed-linked dataset, we generated a timeline for each patient and tracked all their admissions and acute outpatient visits at hospitals across Sweden. Medical records from the different hospitals were either reviewed on location in various electronic medical record systems or obtained on paper copies. We reviewed claims data on location at Löf using the organization’s claims handling software.

### Definitions, inclusion and exclusion criteria

We defined the index admission as the time from patient arrival on the ward to discharge from the ward or the following geriatric or rehabilitation ward. We defined an AE as suffering, physical harm or disease, and death related to the index admission that was not an inevitable consequence of the patient’s disease or treatment. A preventable AE was an event that could have been prevented if adequate actions had been taken during the patient’s contact with healthcare (SFS 2010:659, Swedish Parliament 2010). AEs related to both acts of omission and commission were included.

The inclusion period for all AEs was from the index admission to 90 days postoperatively. We excluded all planned outpatient care and primary care visits. We reviewed 5,422 admissions in 69 hospitals. We included only AEs that were related to index admission. We excluded AEs that were caused during the care for other AEs.

### Review process

The review process has been described in detail in a previous article (Magnéli et al. 2019). We used the Swedish adaptation of the Global Trigger Tool (GTT) (Griffin and Resar 2009), called the Markörbaserad journalgranskning (Sveriges Kommuner och Landsting 2012), a retrospective record review method for identifying AEs. The GTT is a well-studied method that identifies more AEs than other methods (Naessens et al. 2009, Classen et al. 2011). A study-specific manual was created and included all alterations for the GTT.

GTT consists of a 2-stage review process. The first reviewers screened the record searches for any of the 38 predefined triggers indicating a potential AE. In stage 2 the reviewers performed an assessment of potential AEs identified via the triggers and deemed whether they met the inclusion criteria of an AE. The identified potential AEs were assessed regarding causality using a 4-point Likert scale and only included the AEs that were assessed to be caused by the healthcare (those classified as 3 or 4). The severity of the AEs was assessed and classified using a version of the National Coordinating Council for Medication Error Reporting and Prevention (NCC MERP) index (National Coordinating Council for Medication Error and Reporting and Prevention 2001). We included categories E–I. AEs that scored 3 or 4 on the preventability 4-point Likert scale were defined as preventable AEs. The preventable AEs that scored F or more on the NCC MERP index were defined as major preventable AEs.

We performed double review of 6% of the records to assess the agreement of the reviewers in the stage 1 review. We evaluated whether at least 1 trigger or potential AE was identified in the record, whether the record was to be forwarded to secondary review, whether they found the same specific event, and whether this event was a potential AE.

### Patient insurance

According to the Patient Injury Act (Swedish Parliament 2010), the patient has the right to receive compensation if there is predominant probability that the AE was caused by 1 of the following:

1. examination, care, treatment, or similar action provided that the injury could be avoided either by another embodiment of the chosen procedure or by selecting another available procedure which, according to a retrospective assessment, would have satisfied the need for care in a less risky manner;
2. malfunction of a medical device or a medical device used for the examination, care, treatment or similar action or improper handling thereof;
3. incorrect or delayed diagnosis;
4. transmission of infectious agent that led to infection in connection with examination, care, treatment, or similar action;
5. accidents in connection with examination, care, treatment, or similar action or during transport or in connection with fire or other damage to care facilities or equipment;
6. ordering or dispensation of medicines in violation of regulations or instructions.

When examining entitlement to compensation pursuant to the 1st subparagraphs 1 and 3, the standard of action shall apply that applies to an experienced specialist or other experienced professional in the specific field.

### Löf

Löf receives approximately 16,000 claims per year and compensates approximately 40% of those (Löf 2016). Filing a claim is free of charge. Before 2017, the claim to Löf must have been made within 3 years after the AE was noticeable. Löf can compensate for loss of income, other expenses, pain and suffering, and medical invalidity. All assessments are on the individual patient; therefore, the range of compensation, even for the same type of AE, is wide, and study includes too few patients to draw conclusions on compensation levels for different AEs. The experts at Löf uses guidelines from Insurance Sweden in their decision making (Insurance Sweden 2013).

A reviewer (MM) performed the review of Löf’s records (> 5 years after the last surgery in the study and exceeding the 3-year limit for filing claims) and recorded type of injury, reimbursement, and level of disability caused by the AE. All assessments on the claims were made by the experts at Löf and the reviewer only recorded their assessments.

### Statistics

We excluded the non-preventable AEs, because only preventable AEs can be accepted as claims by Löf and also chose to only include the major preventable AEs (NCC MERP index of F or more). We included the claims for AEs within 90 days following surgery that were filed during the 3-year time limit to file claims. We defined confidence intervals (CI) at 95%.

We calculated the claim proportion in the study population with a customized bootstrap function that works as follows: the dataset consists of patient id, sample group (1–20), whether the patient had filed a claim or not and whether the patient had a major preventable AE or not. In each sample group the same number of patients in the group were sampled with replacements. In each group we calculated the filed claim proportion (number of filed claims/number of major preventable AEs). This was multiplied by the group proportion (size of the corresponding group in the study population/study population). The rate times proportion for all 20 groups was summed and this is the point estimate for the claim proportion in the study population. This procedure corresponds to 1 bootstrap repetition and it was repeated 20,000 times. The mean of the samples was the final point estimate and the confidence interval was calculated by using the 2.5th and 97.5th percentiles. For the acute and the elective, the corresponding 10 sample groups were used and for all patients the groups were pooled. The proportion ratio was the mean of the elective patients/mean of the acute patients. We used R (v. 3.5.1; R Project for Statistical Computing, https://www.r-project.org/) for all analyses.

### Ethics, funding, and potential conflicts of interest

Ethical approval was provided by the Regional Ethics Committee of Gothenburg (516-13 and T732-13). The head of each respective unit granted permission for data access for the reviewers. This study was funded by institutional grants from the Karolinska Institutet, Department of Clinical Sciences, Danderyd Hospital, from the regional agreement on medical training and clinical research (ALF) between Stockholm County Council and Karolinska Institutet, and from Löf. None of the authors have any conflicts of interest to disclose.

## Results

### Flow of patients and descriptive data

From the eligible population of 21,774 patients identified in the SHAR, 2,000 patients were included in the study (Figure). 2 patients were excluded from the cohort resulting in 1,998 patients. One did not have an available medical record, and the other did not have hip arthroplasty and was presumed to be a faulty registration in the register. We found 2,116 AEs of different severity in 1,171 (59%) patients. Of these, 1,605 (76%) AEs in 975 (49%) patients were classified as preventable, and 1,066 (50%) in 744 (37%) patients were deemed to be major preventable.

**Figure:**
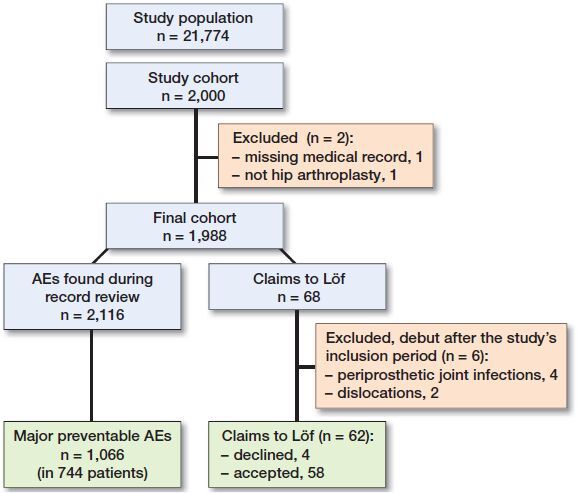
Patient inclusion and exclusions.

There were 144 claims from the patients in the study cohort and 68 of these concerned AEs following hip arthroplasties included in this study. 1 claim was not detected by the record review. 6 claims were excluded from the analysis due to their debut after the study’s inclusion period (4 periprosthetic joint infections [PJIs] and 2 dislocations). 4 patients had their claims declined by Löf because they were not deemed to be preventable AEs, resulting in a 58/62 proportion of accepted claims.

The declined claims included a PJI due to hematogenous spread, a perioperative fracture, pain and numbness of the hands, and a lengthening of the operated leg. Remaining for analysis were 58 claims from 57 patients. The claim patients were younger and consisted of mostly planned surgery patients and total hip arthroplasty patients (Table 3).

### Main results

8% of the patients who sustained a major preventable AE filed a claim with Löf (Table 4). The ratio between the proportion of elective patients/proportion of acute patients was 46.

The total sum of the reimbursement was €296,090, with a mean of €5,220 (range 100–33,860; median = 3,190). The mean grade of disability was 5% (0–40; median = 3) (Table 5).

**Table 5.** All claims and corresponding found AEs

Type of AE	Number of claims n = 62	Accepted claim n = 58	Identified AEs n = 750	Compen-sation mean (€)
Periprosthetic joint infections	37	36	150	5,350
Drop foot and peripheral nerve lesions	9	9	16	5,930
Dislocations	6	6	274	2,780
Pressure ulcers, all categories	1	1	190	4,440
Falls causing fractures	1	1	41	1,100
Perioperative fractures or tissue damage	2	1	33	5,710
Difference in leg length	2	1	20	6,840
Falls causing wounds	1	1	18	620
Implant related AEs **^a^**	1	1	7	20,190
Compartment syndrome	1	1	1	100
Numbness in arms	1	0	0	0

AEs = adverse events.

a Including loosening of implant.

The most common AE type that resulted in a claim was PJI. The PJI patients who filed a claim were younger and consisted of more elective patients, but there was no difference in sex (Table 6). The mean compensation for the 35 PJIs with available compensation data was €5,350 (range 380–33,860, median = 3,430). The mean grade of disability for the PJI patients was 6% (0–40, median = 2.5).

**Table 6.** Demographics of PJI patients. Values are frequency (%) unless otherwise specified

Factor	No PJI patients n = 1,848	PJI patients n = 150	Claim group n = 36	No claim group n = 114
Age, median	77	76	72	77
range	18–100	37–97	37–88	45–97
Female	1,167 (63)	83 (55)	20 (56)	63 (55)
Male	681 (37)	67 (45)	16 (44)	51 (45)
Acute surgery	631 (34)	36 (24)	4 (11)	32 (28)
Elective surgery	1,217 (66)	114 (76)	32 (89)	82 (72)
Total arthroplasty	1,307 (71)	115 (77)	32 (89)	83 (73)
Hemiarthroplasty	541 (29)	35 (23)	4 (11)	31 (27)

PJI = periprosthetic joint infection.

## Discussion

In this cohort study on 1,998 patients undergoing acute and elective hip arthroplasty, we found that only 8% of the 744 patients who sustained a major preventable AE filed a claim with Löf. PJI was the most common AE for filing claims. The proportion of filed claims was almost 50 times higher for the elective patients compared with the acute. In our earlier paper (Magnéli et al. 2019) we found that the 30 days’ incidence of major preventable AEs was more than double for acute patients, compared with the elective (21% vs. 10%). Despite this, only 5 of 62 claims concerned acute surgeries. 58 of the 62 claims were accepted by Löf, suggesting that only obvious AE claims are filed.

### Strengths and limitations

A major strength of this study is the large study cohort and the use of a thorough method for measuring AEs. The study also includes both total and hemiarthroplasties as well as acute and elective surgery, which have not been studied before. This provides a better understanding of the numbers of AEs claimed.

The use of a weighted sample is practical for accumulating high numbers of AEs in a study, but the rates have to be adjusted according to the group weights to represent the proportion in the population. This study includes a wide range of detected AEs of different severity. Many of the minor AEs in the study would likely not result in an accepted claim by Löf. However, the proportion of filed claims for PJI, arguably a severe AE, was only 1 in 4.

### Interpretation and generalizability

Similar to our findings, a recent Swedish study by Kasina et al. (2018) revealed that 25% of the PJIs after total hip arthroplasty filed a claim and that 96% of the claims were accepted by Löf.

Helkamaa et al. (2016) studied filed claims after total hip arthroplasty in Finland, which has a similar patient insurance program to that in Sweden, and found that 44% were accepted, a considerably lower rate than the Swedish studies.

The difference in claims proportion between the acute and elective patients can probably be explained by the fact that these are 2 completely different patient groups and the age difference (median 84 vs. 73) might lead to lower capacity to assimilate information about Löf and act upon it.

PJI was the most common AE for filing a claim. Löf will almost always approve PJI claims. If we hypothesize that all PJIs identified in our study would have been deemed as preventable AEs by Löf, the remaining 114 (76%) PJIs would have received compensation had they filed a claim. Hypothetically, this corresponds to approximately €600,000 of unexploited compensation in this study alone. The incidence rates for PJI are 0.9% after total hip arthroplasty (Lindgren et al. 2014) and 5–6% for hemiarthroplasty (de Jong et al. 2017, Guren et al. 2017). In 2015, over 16,000 total hip arthroplasties and 4,200 hemiarthroplasties were performed in Sweden, which would hypothetically generate 350 to 396 PJIs per year, with 263 to 297 of these not filing a claim. This would correspond to €1.4– 1.6 million of unexploited compensation each year.

## Conclusion

The proportion of filed claims for major preventable AEs following hip arthroplasty is very low in Sweden. This is true even for obvious and serious AEs such as periprosthetic joint infection. The proportion of filed claims is higher for elective than acute patients. Whether the healthcare system fails to inform patients about their right to claim for compensation or the patients are informed but choose not to file a claim is unknown.

## Supplementary data

Tables 1 and 2 are available as supplementary data in the online version of this article, http://dx.doi.org/10.1080/17453674.2019.1677382

## Supplementary Material

Supplemental Material
