# Supplementary material for: Only 8% of major preventable adverse events after hip arthroplasty are filed as claims: a Swedish multi-center cohort study on 1,998 patients
Source: Acta Orthop. 2019 Oct 16;91(1):20–5. doi: 10.1080/17453674.2019.1677382 (PMC7006721; doi:10.1080/17453674.2019.1677382)
Supplement: Supplemental Material [file IORT_A_1677382_SM6628.pdf]

Supplementary data

Table 1. Selection groups used for the weighted sample

	Acute		Elective	
	Population	Sample	Population	Sample
**With a predefined ICD-10 code indicating an AE in the NPR**				
Percentiles of length of stay				
0–55%	194	11	95	22
56–80%	148	16	58	33
81–100%	302	25	235	49
Readmission				
2–30 days	274	98	356	196
31–90 days	199	98	204	195
**Without a predefined ICD-10 code indicating an AE in the NPR**				
Percentiles of length of stay				
0–55%	2,859	44	9,769	86
56–80%	1,167	65	2,070	131
81–100%	766	97	1,781	197
Readmission				
2–30 days	294	147	337	295
31–90 days	341	66	325	129
Total	6,544	667	15,230	838

ICD-10 = the 10th revision of the International Classification of Diseases (WHO 2017).
NPR = National Patient Register.

Table 2. Set of ICD-10 codes used in the selection of patients

**As main diagnosis**	
All I codes	Diseases of the circulatory system
J819	Pulmonary edema
J13	Pneumonia due to Streptococcus pneumoniae
J15	Bacterial pneumonia, not elsewhere classified
J18	Pneumonia, organism unspecified
R33	Retention of urine
**As main or secondary diagnosis**	
I803	Phlebitis and thrombophlebitis of lower extremities, unspecified
I269	Pulmonary embolism without mention of acute cor pulmonale
L899	Decubitus ulcer and pressure area, unspecified
M243	Pathological dislocation and subluxation of joint, not elsewhere classified
M244	Recurrent dislocation and subluxation of joint
S730	Dislocation, sprain and strain of joint and ligaments of hip
T810	Hemorrhage and hematoma complicating a procedure, not elsewhere classified
T813	Disruption of operation wound, not elsewhere classified
T814	Infection following a procedure, not elsewhere classified
T840	Mechanical complication of internal joint prosthesis
T845	Infection and inflammatory reaction due to internal joint prosthesis
T933	Sequelae of dislocation, sprain and strain of lower limb

ICD-10, see Table 1
